# Music informer as an efficient model for music generation

**DOI:** 10.1038/s41598-025-02792-4

**Published:** 2025-06-06

**Authors:** Hui Sun, Xiaofang Wang, Yuxing Wang, Pengfei Lu

**Affiliations:** College of Information Science and Technology, Shihezi University, Xinjiang, 832003 China

**Keywords:** Music generation, Music informer, Music transformer, Informer, Attention mechanism, Computational science, Computer science

## Abstract

Music Transformer has been extensively employed in music generation, however, the self-attention mechanism consumes significant memory due to its complexity. To address this issue, Music Informer is proposed, drawing inspiration from the widely-used Informer model in fields like time series forecasting, weather prediction, etc. Music Informer primarily utilizes the ProbSparse self-attention mechanism, relative local attention mechanism, and LSTM structure. Objective results indicate that Music Informer conserves 21.73%, 31.87%, and 41.33% of computational resources compared to Music Transformer, Performance RNN, and Multi-Track Music Transformer under identical experimental conditions. Additionally, music samples generated by Music Informer outperform those from the three baselines in the metrics of Pitch Class Entropy, Number of Pitch Classes, Pitch Entropy, Number of Pitches, Average Inter-Onset Interval. Furthermore, Music Informer demonstrates higher Overlap Area values in the features Number of Pitches, Pitch Class Entropy, Average Inter-Onset Interval, Groove Consistency, and Pitch Entropy, as well as lower Kullback–Leibler Divergence in Average Inter-Onset Interval and Groove Consistency, highlighting a strong alignment with the feature distribution of the real dataset for these specific characteristics. Subjective results suggest that listeners prefer music generated by Music Informer over the baselines due to its improved coherence and overall quality.

## Introduction

Artificial intelligence is progressively infiltrating the music industry. Research on deep learning models in music spans various aspects, including polyphonic music generation^1^, accompaniment generation^2^, style transfer^3^, music recommendation^4^, and more. These models generate smoother and more aesthetically pleasing music by bypassing the need for decision-making processes or strict adherence to specific music styles during generations. Additionally, deep learning-based models empower creators to produce high-quality music without extensive knowledge of music theory^5,6^.

Initially, music generation did not predominantly rely on neural network techniques but was instead rooted in data-driven statistical methods such as the N-gram model^7^ and Markov model^8^. Rule-based music generation methods^5^ also existed, utilizing specific rules or grammar for composing music. However, these models were limited in creativity, flexibility, musical expression, and adaptability.

In recent years, significant advancements have been made in sequence-based music generation. Early works by Klara^9^ and Oore^10^ were among the first to represent music content as symbolic sequences, subsequently input into neural network models for training. Prominent examples of these models include those based on Recurrent Neural Networks (RNNs)^11^, Long Short-Term Memory (LSTM)^12,13^, Generative Adversarial Networks (GAN)^14,15^, and Transformer^16,17^. Shi et al. proposed an emotion-based music generation model built on the C-RNN-GAN foundation to generate emotional music^11^. Li et al. introduced a Xi’an drum music generation method using Bi-LSTM deep reinforcement learning to generate Xi’an drum music^18^. Due to the effectiveness of the Transformer, which captures long-range dependencies in input sequences^19,20^ , Huang et al. introduced Music Transformer for symbolic music generation in 2018^21^. Huang et al. also proposed Pop Music Transformer, excelling in both subjective and objective evaluations^22^. Aashiq et al. developed Transformer-GAN, capable of generating long music sequences exceeding 1000 tokens, performing comparably to the Music Transformer^23^. Di et al. optimized Music Transformer for composing background music for videos, producing melodious and compatible music based on the given video^24^. Pedro Sarmento et al. proposed Transformer-XL that can generate guitar tablature based on the desired instrument or the desired music genre, with successful outcomes in respective control tasks^25^. Zhang et al. introduced Harmony-Aware Hierarchical Music Transformer, a model that dynamically incorporates musical structure to refine the structuring of diverse musical elements across multiple levels^26^. Hsiao et al. improved the modeling approach with Compound Word Transformer^27^, using Linear Transformer^28^ as its underlying architecture and can generate multiple tokens at each time step. Dong et al. further explored multitrack music modeling with compound words, proposing Multitrack Music Transformer to address the long sequence problems in existing multitrack music representations^29^. Shih et al. proposed Theme Transformer which can generate polyphonic pop piano music with reasonable variations and repetitions based on given conditions^30^. Huang et al. proposed Hyperbolic Music Transformer, encoding music sequences into the hyperbolic space for structured music generation^31^. Wang et al. introduced Motif transformer to generate music with motifs^32^.

While these Transformer-based models improve the quality of the generated music to some extent, the complexity of the self-attention mechanism and memory usage require substantial computing resources and extended training times.

Informer^33^ adopts the ProbSparse self-attention mechanism, achieving efficient time complexity. This advancement significantly enhances sequence dependency alignment, resulting in more reliable and accurate performance. With the capability to handle long sequences of data and capturing global dependencies, Informer is widely used in fields such as time series forecasting, weather forecasting, energy load forecasting, and traffic flow prediction.

Recognizing music as a temporal sequence, Informer demonstrates significant potential for music generation by efficiently handling extensive music data sequences, capturing long-term dependencies, and generating diverse musical compositions. Consequently, this study introduces an efficient music generation model, named Music Informer. The music generated by this model received high evaluations from participants, demonstrating strong coherence and overall quality. Notably, Music Informer boasts lower memory usage complexity compared to the Music Transformer and other two models used for music generation.

## Materials and methods

### Materials

In symbolic music generation, various datasets are available in formats such as MIDI, MusicXML, and ABC notation^34^. This article selects the MAESTRO dataset^35^ for its substantial size, diversity, quality and popularity in the field. Comprising approximately 200 h of paired audio and MIDI recordings from a decade of the International Piano-e-Competition, the dataset includes detailed MIDI data with key strike velocities and sustain/sostenuto/una corda pedal positions. The audio and MIDI files are precisely aligned within an accuracy of about 3 ms and are segmented into individual musical pieces, each annotated with the composer, title, and year of performance.

This dataset is divided into training, testing, and validation sets in an 8:1:1 ratio and is available for download from the MAESTRO dataset website: https://magenta.tensorflow.org/datasets/maestro.

### Methods

This section delineates the research methodology employed in this paper, encompassing data processing, model construction, and model evaluation.

### A. Data processing

Musical Instrument Digital Interface (MIDI)^36^ is an industry standard that defines an interoperability protocol between various electronic instruments, software, and devices. This standard simplifies the interconnectivity of products from diverse companies, encompassing digital devices and a myriad of other products. Thanks to the advantage of midi files such as flexibility, compactness and customizability, musicians and a significant number of arrangers use MIDI to create and share their music works.

**Fig. 1:**
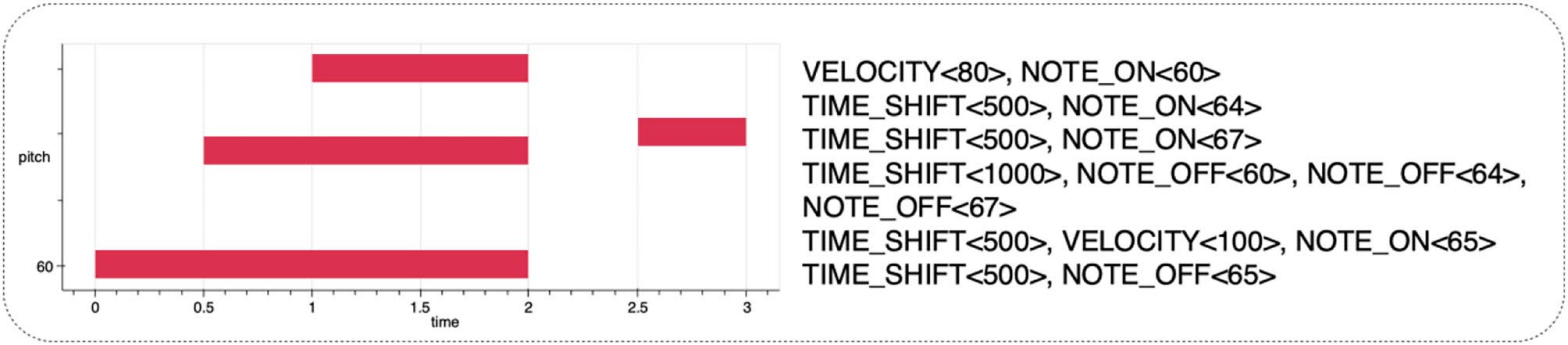
An example of MIDI-Like representation. A C Major chord is played at a velocity of 80 and sustained until the 2-s mark, followed by an F note with a velocity of 100 for 0.5 s.

MIDI transmits notes, control parameters, and other instructions, with NOTE_ON and NOTE_OFF being extensively utilized for music representation. Consequently, many researchers employ MIDI-Like^6^ events to represent music, a method known as event-based music representation. Figure 1 illustrates the MIDI-Like representation, where a piano performance is visually presented as a piano-roll diagram (depicted on the left) and encoded as a series of events (depicted on the right). This encoding scheme employs a vocabulary comprising NOTE_ON events, NOTE_OFF events, TIME_SHIFT events, and VELOCITY events. Table 1 shows the vocabulary.

**Table 1:** Vocabulary of the MIDI-Like representation. NOTE_ON events indicates the start of a note, with a range of [0, 128]. NOTE_OFF events indicates the end of a note, with a range of [128, 256]. TIME_SHIFT events represents the time interval between notes, with a range of [256, 356]. VELOCITY events indicate the strength or intensity of note playing and are divided into 32 levels of intensity.

Events	Description	Range
NOTE_ON	A specific note starts playing	[0, 128]
NOTE_OFF	A specific note stops playing	[128, 256]
TIME_SHIFT	The time interval or delay between notes	[256, 356]
VELOCITY	The strength or intensity of a note	[356, 388]

**Fig. 2:**
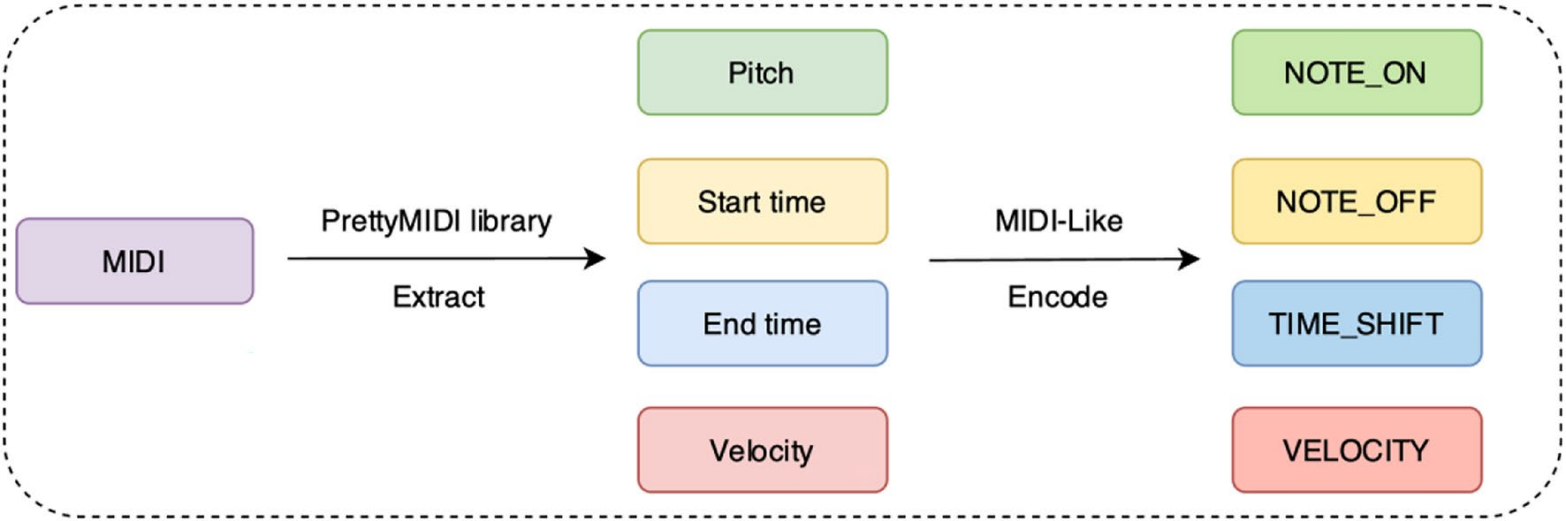
The process of handing MIDI files.

Using the PrettyMIDI library, data from MIDI files in the MAESTRO dataset is read, extracting the pitch, start time, end time, and velocity of each note. Subsequently, the MIDI-Like representation is applied to encode the extracted content. Figure 2 illustrates the process of handling MIDI files.

### B. Model construction

Music Informer builds upon the fundamental Informer but utilizes only the decoder. In music generation, the goal is to create a new sequence rather than transform an input sequence into another form or extract information. Using only the decoder simplifies the structure of the model and training process. Since complex input-to-output mappings are unnecessary in music generation, excluding the encoder reduces the number of model parameters and decreases training complexity while maintaining the quality of the generated music.

**Fig. 3:**
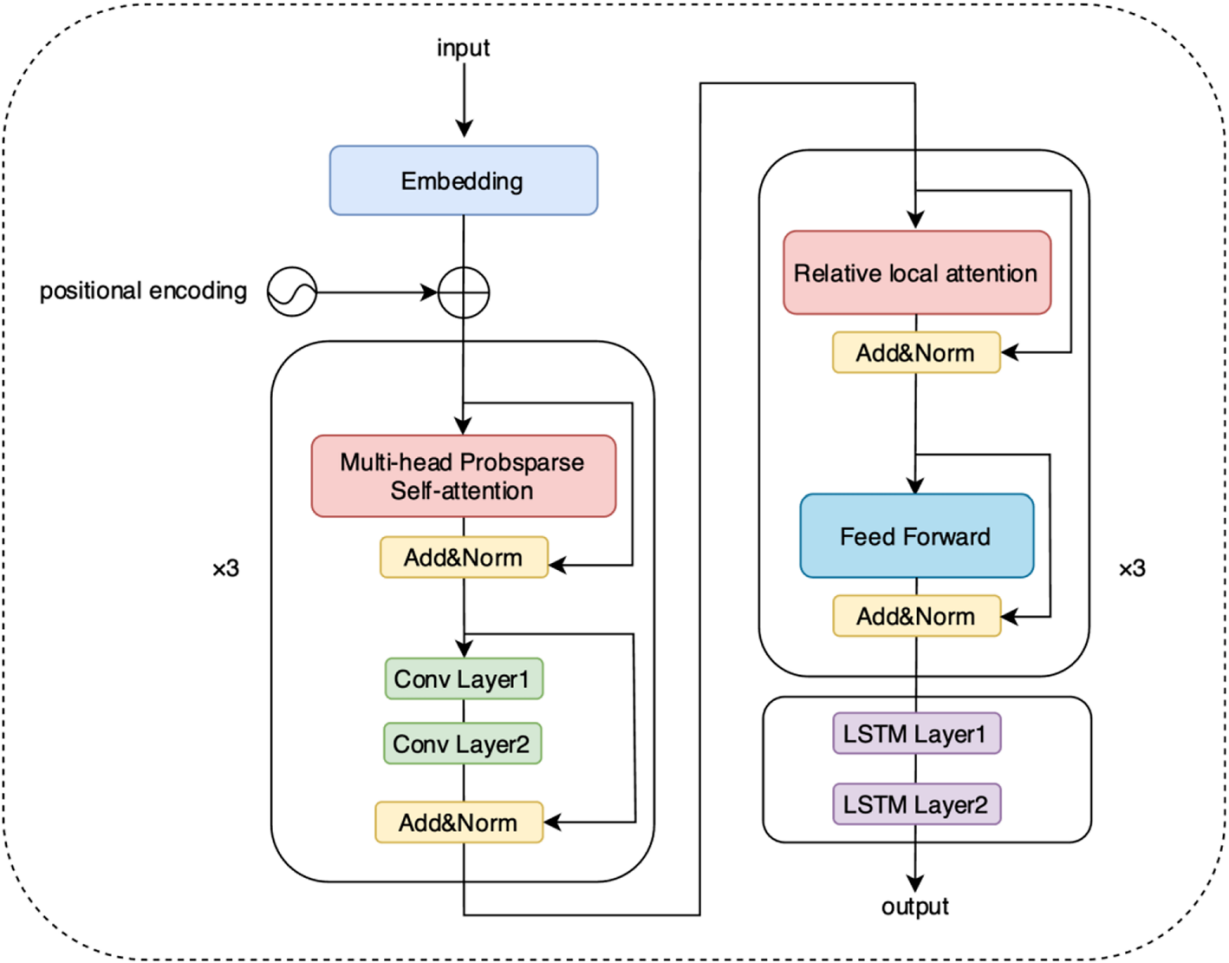
The structure of the Music Informer. The model is composed of three ProbSparse self-attention layers, three relative local attention layers, and an LSTM structure.

The decoder structure of Music Informer comprises an embedding layer, a positional encoding layer, and three layers each of ProbSparse self-attention and relative local attention, totaling six layers. Following the decoder is an LSTM structure with two LSTM layers, which ultimately produces the final output. Figure 3 illustrates the model architecture.

Given the MIDI-Like word *M* $\in {\mathbb{R}}^{n}$, the feature matrix $X\in {\mathbb{R}}^{n}$ is derived by integrating the positional embedding with the word embedding. The positional encoding formulas are provided below:

1 $$\left\{ {\begin{array}{*{20}c} {{\text{PE}}\left( {pos,2i} \right) = \sin \left( {\frac{pos}{{10000^{{\frac{2i}{{d_{{{\text{model}}}} }}}} }}} \right), } \\ {PE\left( {pos,2i + 1} \right) = \cos \left( {\frac{pos}{{10000^{{\frac{2i}{{d_{{{\text{model}}}} }}}} }}} \right),} \\ \end{array} } \right.$$

where ${d}_{\text{model}}$ represents the dimension of the vector after word embedding, $pos$ represents the position of elements in the sequence, $i$ represents the index in the positional encoding. The computation for the embedding layer and the positional encoding can be expressed as shown in Eq. (2).

2 $$X=\text{Emb}\left(M\right) + \text{PositionEnc}\left(M\right).$$

After the positional encoding, the matrix $X$ undergoes a matrix multiplication to be mapped into the attention space through:

3 $$\begin{gathered} Q = XW_{{\text{Q}}} , \hfill \\ K = XW_{{\text{K }}} , \hfill \\ V = XW_{{\text{V }}} , \hfill \\ \end{gathered}$$

where ${W}_{\text{Q}}$, ${W}_{\text{K}}$, and ${W}_{\text{V}}$ represent three different weight matrices, which linearly transform the input matrix $X$ into $Q$, $K$, and $V$, respectively.

ProbSparse Self-attention introduces probabilistic sparsity by selectively focusing on a subset of elements within the input sequence. This approach aims to enhance computational efficiency and model performance. It involves calculating similarity scores between input elements and selecting participants based on a predetermined probability threshold or distribution. Subsequently, attention weights are computed using the Softmax function, and the final self-attention output is derived through weighted summing. The calculation formula for ProbSparse Self-attention is as follows:

4 $$Z = {\text{A}}\left( {Q,K,V} \right) = {\text{Softmax}}\left( {\frac{{\overline{Q}K^{{\text{T}}} }}{\sqrt d }} \right)V,$$

where $d$ represents the dimension of $Q$ and $K$, the sparse matrix $\overline{Q }$ contains only the Top-u queries under the sparsity measurement, u is set to the constant sampling factor c times the natural logarithm of with ${L}_{\text{Q}}$ representing the length of $Q$. This allows ProbSparse self-attention to compute only $\mathcal{O}(\text{ln}{L}_{\text{Q}})$ dot products for each query-key lookup, resulting in memory usage within the layer of ${\mathcal{O}}{\text{(L}}_{{\text{K}}} \;{\text{ln}}\;{\text{L}}_{{\text{Q}}} {)}$, where ${L}_{\text{K}}$ represents the length of $K$. Under multi-head attention, this mechanism generates different sparse query-key pairs for each head, thereby avoiding significant information loss.

The output of ProbSparse Self-attention is followed by a fully connected feedforward neural network, with residual connections and normalizations calculations at

5 $$L_{{\text{m}}} = {\text{LayerNorm}}\left( {Z + X} \right).$$

Then, the vector ${L}_{m}$ is fed into two convolutional layers, followed by a fully connected feedforward neural network. Residual connections and normalization calculations are applied throughout the network at

6 $$L_{1} = {\text{LayerNorm}}\left( {{\text{Conv}}2\left( {{\text{Conv}}1\left( {L_{{\text{m}}} } \right)} \right) + L_{{\text{m}}} } \right).$$

**Fig. 4:**
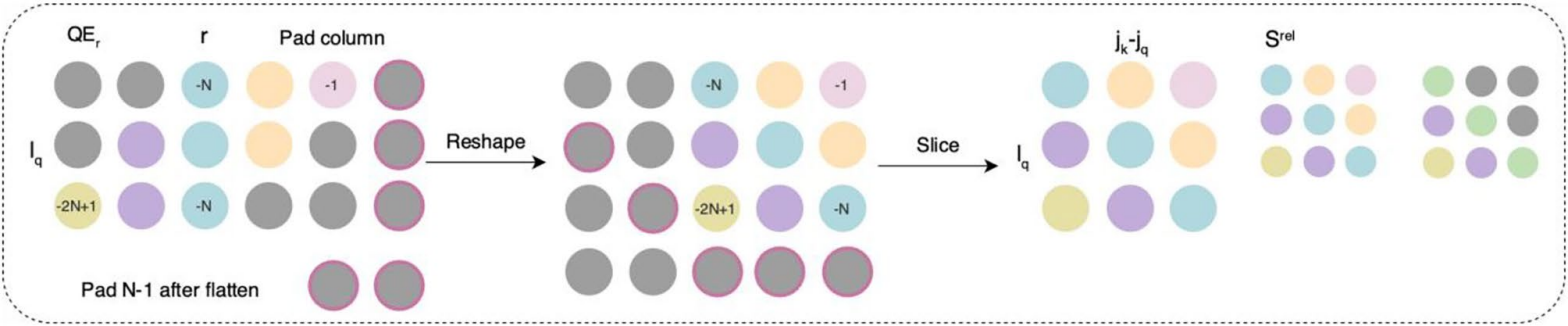
Relative local attention^21^: The thumbnail on the right demonstrates the intended arrangement for ${S}^{\text{rel}}$.

Relative local attention, introduced by Shaw^37^, directs attention based on the distance between two positions in a sequence. In music generation and processing, capturing and utilizing local features is crucial for generating music with flexibility and nuance. Relative attention mechanisms enhance a model’s ability to understand and manage these local features. This process involves learning a separate relative position embedding ${E}_{r}$ with a shape of $(H,L,D)$, where $H$ represents the number of attention heads, $L$ signifies the sequence length, and $D$ indicates the embedding dimension. This embedding encompasses representations for all potential relative distances $r={j}_{\text{k}}-{i}_{\text{q}}$ between a query position ${i}_{\text{q}}$ and a key position ${j}_{\text{k}}$. These embeddings are structured by distance from − *L* + 1 to 0 and are trained individually for each attention head. These relative embeddings interact with the queries, generating an output ${S}^{\text{rel}}$ just as shown in Fig. 4. The calculation formula of relative local attention is as follows:

7 $${\text{RelativeAttention}} = {\text{Softmax}}\left( {\frac{{QK^{{\text{T}}} + S^{{{\text{rel}}}} }}{\sqrt d }} \right)V.$$

The vector ${L}_{1}$ is then fed into the relative attention layer, and the results are passed into a fully connected feedforward neural network with residual connections and normalization. The calculation can be represented as follows:

8 $$L_{2} = {\text{RelativeAttention}}\left( {L_{1} } \right),$$

9 $$L_{3} = {\text{LayerNorm}}\left( {L_{1} + L_{2} } \right),$$

10 $$L_{4} = {\text{LayerNorm}}\left( {L_{3} + {\text{FeedForward}}\left( {L_{3} } \right)} \right).$$

RNNs can process sequential data and extract temporal and semantic information, achieving significant results in areas such as speech recognition and music generation. However, they often encounter gradient vanishing and gradient explosion when handling long sequence tasks^6^. To address these issues, the LSTM network was proposed. LSTMs consist of four components: the forget gate, the input gate, the cell state, and the output gate^38^. These components collaborate to determine whether information remains usable; information deemed usable after algorithmic evaluation is retained, while unusable information is discarded through the forget gate.

A two-layer LSTM was employed after the decoder to further process and learn the temporal dependencies and complex structures in the music, ultimately producing the final output. The calculation formula for this process is as follows:

11 $$Y = {\text{LSTM}}\left( {{\text{LSTM}}\left( {L_{4} } \right)} \right).$$

**Model setting** Music Informer builds on the architecture of Music Transformer, retaining key components such as the relative attention mechanism. To enhance performance, we introduce the ProbSparse Self-attention mechanism, LSTM, and convolutional layers, which reduce computational complexity during training while achieving higher accuracy and lower training loss. Our model was implemented using the PyTorch framework and consisted of a 6-layer encoder with 8 attention heads, a hidden layer dimension of 512, and a feedforward layer dimension of 1024. To alleviate gradient vanishing issues, we used the ReLU activation function, and subsequently optimized the model utilizing the Adam optimizer. The LSTM structure comprised two LSTM layers with a feature dimension of 512 and a hidden layer dimension of 1024. To prevent overfitting, we set the dropout parameter to 0.1. The model was trained for 200 epochs on an NVIDIA A100 GPU with a batch size of 2. In all experiments, we utilized beam search with a beam size of 0 and employed random sampling from the probability distribution. We used Music Transformer, Performance RNN^39^ and Multitrack Music Transformer^29^ as baselines, and all of these models were trained on the same MAESTRO Dataset.

### C. Model evaluation

#### Objective evaluation

To validate the effectiveness of the proposed model, an objective evaluation was conducted using cross-entropy loss and accuracy as the primary metrics for model training and assessment. Furthermore, both absolute measurement and relative measurement were employed to evaluate the melodic quality of the generated samples.

**Absolute measurement** The absolute measurement evaluates the attributes and features of datasets, enabling comparison of attribute differences between the training dataset and the generated data, or feature differences between data generated by two different models. For this purpose, MusPy, a tool developed by Dong et al.^40^, was utilized for feature extraction and evaluation of model-generated samples. The evaluation metrics include:

Pitch Range (PR): Pitch range is defined as the interval between the lowest and highest notes in a piece of music. It reflects the overall span and diversity of pitch content in the composition.

Pitch Class Entropy (PCE): Entropy of the histogram of the pitch class in the standard tone scale. This metric involves constructing a 12-dimensional pitch class histogram $\overrightarrow{h}$ based on the notes’ pitch classes, normalized by the total note count in the piece to sum to 1. The entropy of $\overrightarrow{h}$ is then calculated to quantify its distribution as (12):

12 $$H\left( {\vec{h}} \right) = {-}\mathop \sum \limits_{i = 0}^{11} h_{{\text{i}}} {\text{log}}_{2} h_{{\text{i}}} ,$$

where ${h}_{\text{i}}$ represents the normalized frequency of the i-th pitch class.

Groove Consistency (GC): Groove consistency describes the repetitiveness and uniformity of rhythmic patterns in a musical time series, reflecting the stability and regularity of the rhythm. The calculation formula is as follows:

13 $$GC\left( {\vec{g}^{{\text{a}}} ,\vec{g}^{{\text{b}}} } \right) = 1{-}\frac{1}{Q}\mathop \sum \limits_{i = 0}^{Q - 1} {\text{XOR}}\left( {g_{{\text{i}}}^{{\text{a}}} ,g_{{\text{i}}}^{{\text{b}}} } \right),$$

where ${\overrightarrow{g}}^{\text{a}}$ and ${\overrightarrow{g}}^{\text{b}}$ represent two different rhythm patterns, $Q$ denotes the length of the rhythmic pattern, ${g}_{\text{i}}^{\text{a}}$

and ${g}_{\text{i}}^{\text{b}}$ represent ryhthmic events at the i-th time point.

Number of Pitch Classes (NPC): This metric counts the distinct pitch classes in a music piece, ignoring any octave differences.

Pitch Entropy (PE): Pitch entropy evaluates the uniformity of pitch distribution, considering specific pitches along with their octave information.

Number of Pitches (NP): This parameter tallies the different pitches within a musical work, providing insight into the pitch range and complexity. Higher values often denote greater complexity and pitch variation.

Chord Histogram Entropy(CHE): This measure assesses the uniformity of chord usage^41^. A higher CHE value signifies more varied and diverse chord usage, indicating greater complexity and innovation in harmonic treatment within a composition.

Average Inter-Onset Interval (AIOI): The average inter-onset interval quantifies the time interval between consecutive notes, aiding in the analysis of rhythm and overall musical structure by describing the temporal distribution of notes.

**Relative measurement** Relative measurement assesses the similarity of generated samples to a reference dataset. MGEval, a toolbox designed by Yang^42^, is utilized for the objective as assessment of music generation. It derives features rooted in pitch and duration to quantify how closely the generated music adheres to the statistical patterns of the training dataset. These features are framed as probabilistic distributions, enabling the evaluation of the generation model’s performance through relative metrics, which compare two distributions across various dimensions.

Initially, a leave-one-out cross-validation technique is employed to compute the distances between each sample and the remaining instances within the same dataset, creating a distance histogram specific to each feature. Subsequently, these histograms undergo smoothing through Kernel Density Estimation (KDE), a process that approximates the probability density function (PDF) of each feature histogram.

**Fig. 5:**
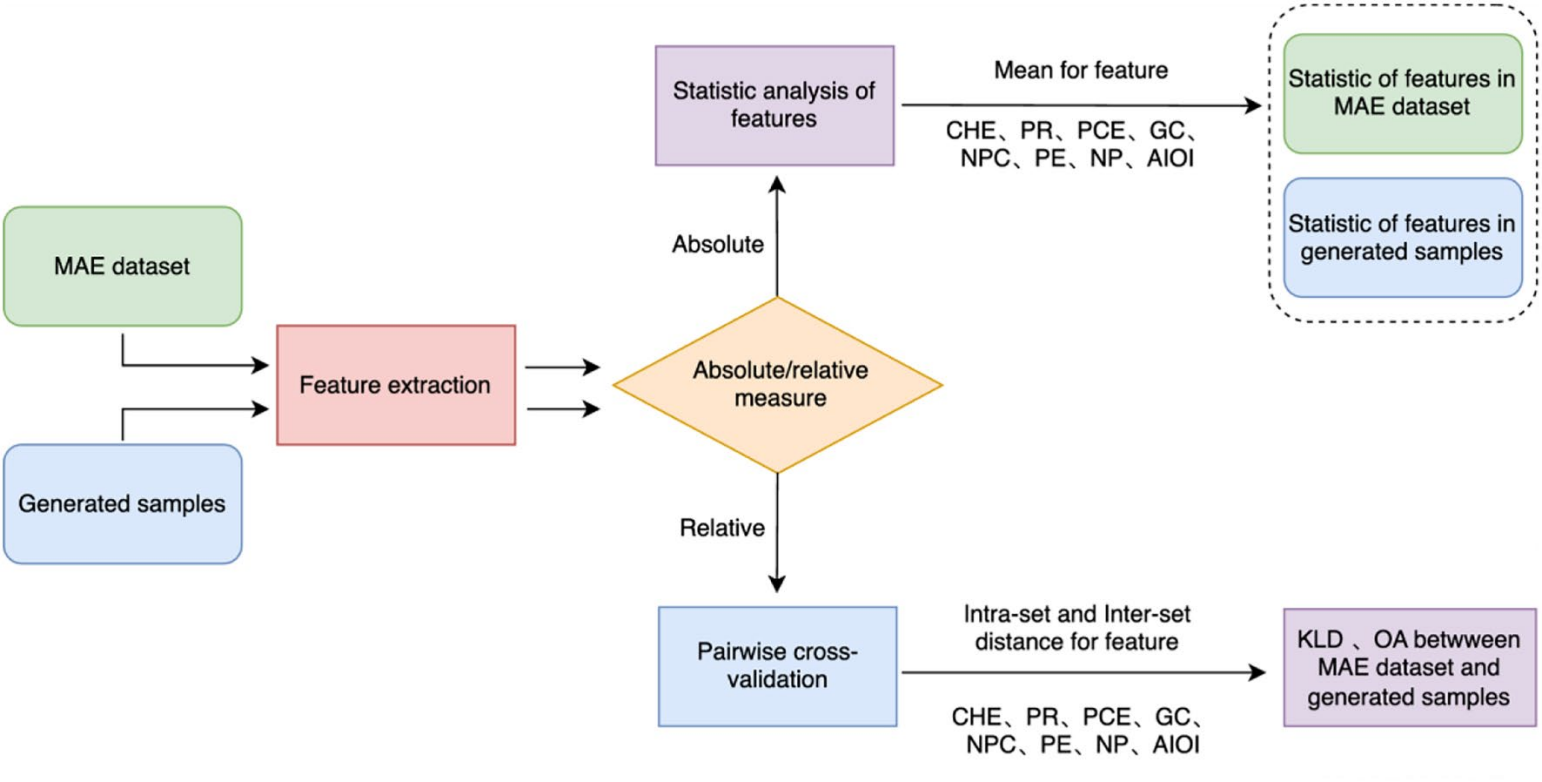
Process of relative measurement and absolute measurement for models.

The resulting PDFs facilitates the calculation of the Overlap Area (OA) and Kullback–Leibler Divergence (KLD). These metrics measure similarity, indicating whether the model has effectively learned the features of the training dataset. Specifically, a smaller KLD and a larger OA between the inter-set distance of two datasets and the intra-set distance of the MAESTRO dataset suggest a higher similarity to the MAESTRO dataset. The process diagram for absolute measurement and relative measurement is shown in Fig. 5:

#### Subjective evaluation

In addition to objective evaluations of the generated samples, subjective evaluations were also conducted.

The musical compositions produced were evaluated utilizing a 5-point grading system, ranging from 1 to 5, with higher scores reflecting superior performance. This assessment encompassed three primary dimensions: Coherence (C), Naturalness in Pitch (N), and Overall Quality (Q) ^43^, each of which contributed to an overall rating that reflects the composition’s excellence.

Objective evaluations offer quantifiable metrics, whereas subjective evaluations capture users’ feelings and perceptions, enhancing the comprehensiveness and reliability of the evaluation results. The combination of objective and subjective evaluation allows for a holistic assessment of the proposed model’s effectiveness and performance in music generation tasks.

## Results

The experimental results are categorized into objective and subjective results. Objective results reflect the model’s performance and the quality of the generated samples, providing metrics to assess the accuracy and reliability of the model. Conversely, subjective results emphasize human perception and aesthetic experience, evaluating whether the generated music exhibits emotional expression, creativity, and artistic value. By integrating objective results and subjective results, a comprehensive assessment of the model’s performance and quality is achieved.

### A. Objective results

The objective results are categorized into three parts: training results, absolute measurement results, and relative measurement results.

**Fig. 6:**
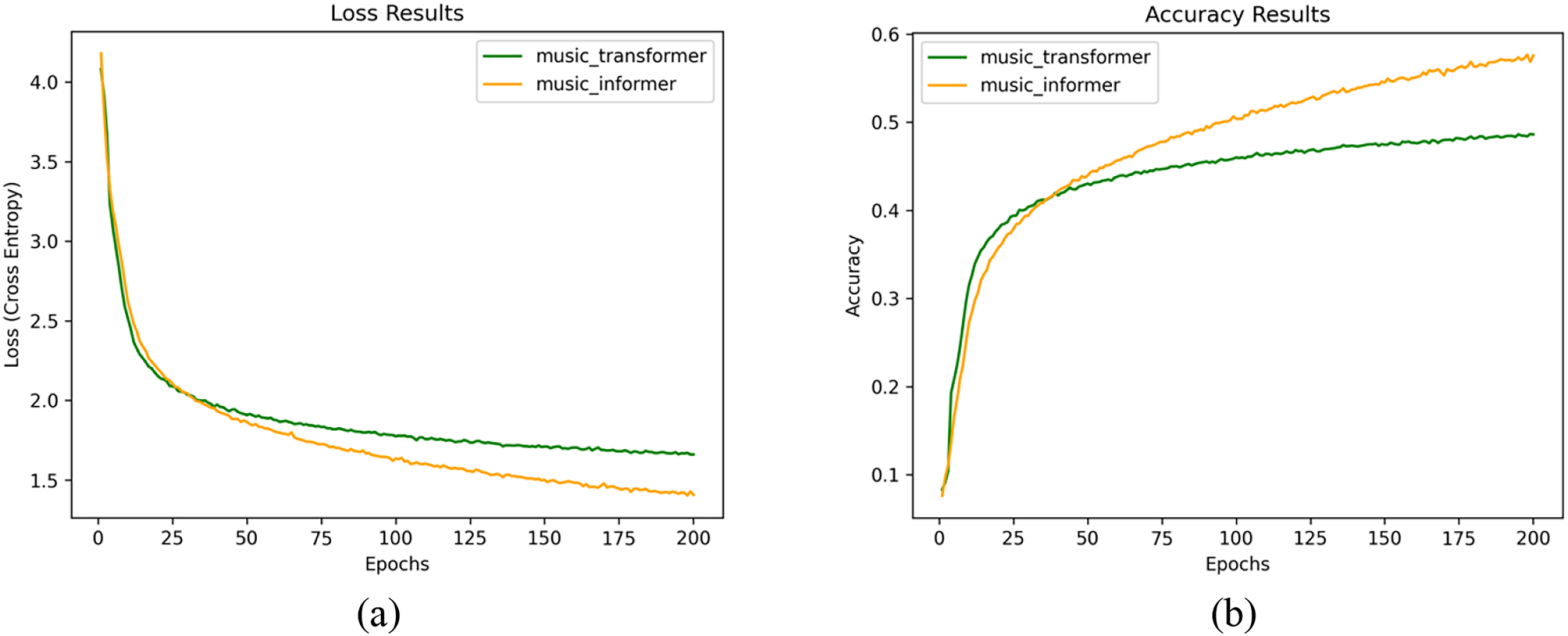
(**a**) Loss curve accuracy curve of Music Informer and Music Transformer. (**b**) Accuracy curve of Music Informer and Music Transformer.

**Fig. 7:**
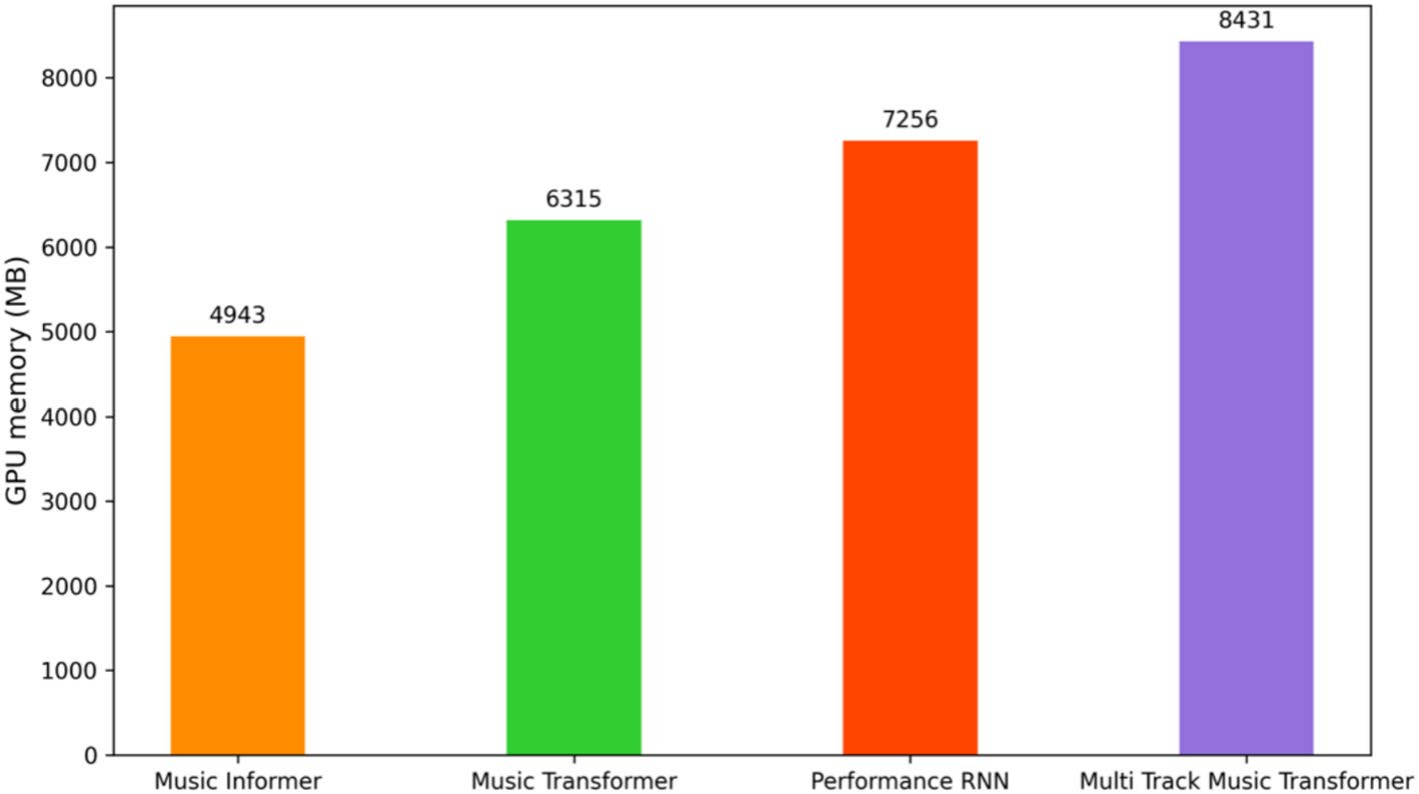
GPU memory usage of Music Informer, Music Transformer, Performance RNN, Multi-track Music Transformer based on the experimental conditions detailed in the model setting section.

**Training results** The training results of the Music Informer model on the MAESTRO dataset are illustrated in Fig. 6, which presents the model’s loss curve and accuracy curves. To further evaluate the model’s efficiency, we also provide the model’s memory usage. Figure 7 shows that, based on the experimental conditions outlined in the model setting section, the Music Informer utilized 4943 MB of GPU memory. This consumption is lower than that of the other three models and results, resulting in significant savings in computational resources. This efficiency is likely due to the probabilistic self-attention mechanism, which selectively focuses on a subset of elements in the input music sequence.

**Absolute measurement results** MusPy is utilized to evaluate the generated samples of the model based on specific metrics. For the MAESTRO dataset, these metrics are calculated for all samples, and the averages are taken. Music Informer, along with three other baseline models, each randomly generates 500 MIDI samples, from which features are extracted, metrics calculated, and averages obtained. The comparison of objective metrics between all models and the real dataset is presented in Table 2. It can be observed that our model surpasses the other three models in metrics such as PCE, NPC, PE, NP, and AIOI, achieving favorable results. This demonstrates that our model is capable of generating compositions that are closer to the dataset. In contrast, Performance RNN excels in the PR and GC metrics. Although Music Informer underperforms the baseline models (Music Transformer and Performance RNN) on the CHE metric, its higher CHE score still suggests a potential to generate samples with more complex and diverse harmonic structures.

**Table 2:** Objective metric results for the MAESTRO dataset, music informer, music transformer, performance RNN, and multi-track music transformer.

Model\Metrics	CHE	PR	PCE	GC	NPC	PE	NP	AIOI
MAESTRO dataset	4.2408	68.1817	3.3454	0.7473	11.9898	5.3777	66.0468	0.1082
Music transformer	**4.5422**	58.4927	3.1463	0.7837	11.7257	4.8744	46.6019	0.1327
Performance RNN	4.5529	**61.1918**	3.2978	**0.7558**	11.5134	5.0714	49.3316	0.1490
Multi-track music transformer	3.3390	54.06	2.5429	0.8360	11.08	2.5362	38.02	0.1394
Music informer	5.1391	58.6129	**3.3171**	0.7630	**11.9483**	**5.1511**	**50.5935**	**0.1183**

The bold indicates the optimal objective results for different metrics under different models compared with the MAESTRO dataset

**Fig. 8:**
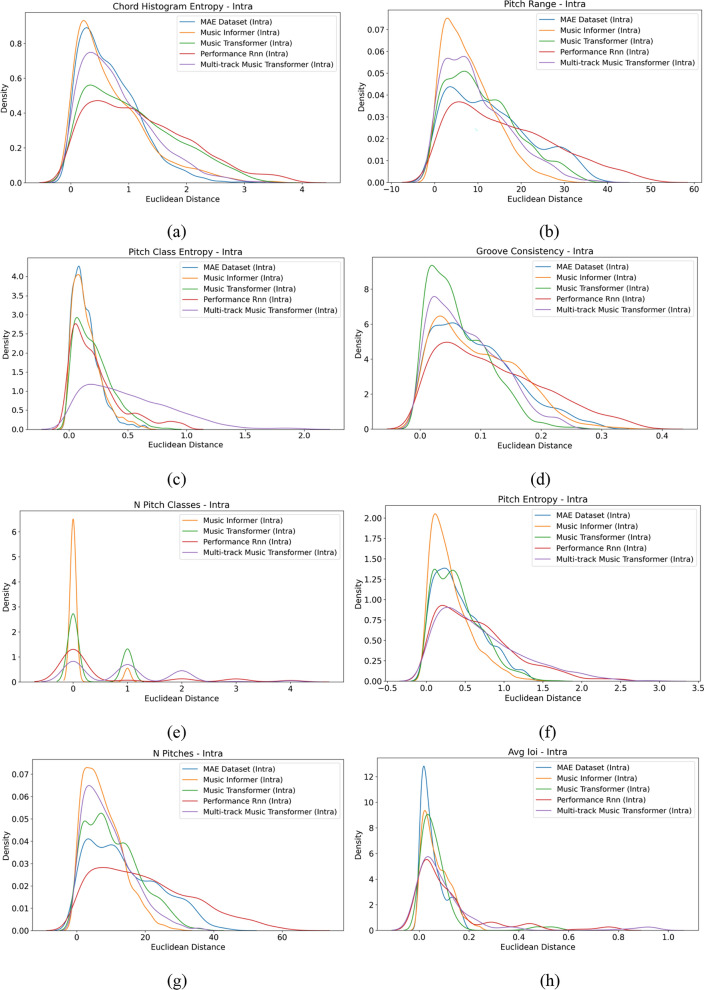
An example of intra-set distance for MAESTRO Dataset, Music Informer, Music Transformer, Performance RNN, and Multi-track Music Transformer. The metrics displayed are: (**a**) CHE, (**b**) PR, (**c**) PCE, (**d**) GC, (**e**) NPC, (**f**) PE, (**g**) NP and (**h**) AIOI.

**Relative measurement results** The MGEval tool was employed to evaluate the samples. Fifty samples were randomly selected from those generated by the MAESTRO dataset, Music Informer, and three baseline models. Features CHE, PR, PCE, GC, NPC, PE, NP, and AIOI were extracted from these samples, followed by a relative evaluation. The histograms of Euclidean intra-set distances for these features from both the dataset and model-generated samples are displayed in Fig. 8. In these histograms, the horizontal axis represents Euclidean distance, while the vertical axis represents density. As shown in the graph, Music Informer exhibits a distinct peak within a certain range for the metrics CHE, PR, PCE, GC, NPC, PE, NP, and AIOI, indicating relatively stable results. It is worth noting that the MAESTRO dataset has a fixed NPC feature value of exactly 12 for all samples. Consequently, the Euclidean distance for this feature is consistently zero, resulting in a single-point distribution with no variation to visualize. This uniformity in the MAESTRO dataset prevents it from being represented in the density plot, as density plots are designed to illustrate variations in data distributions.

**Fig. 9:**
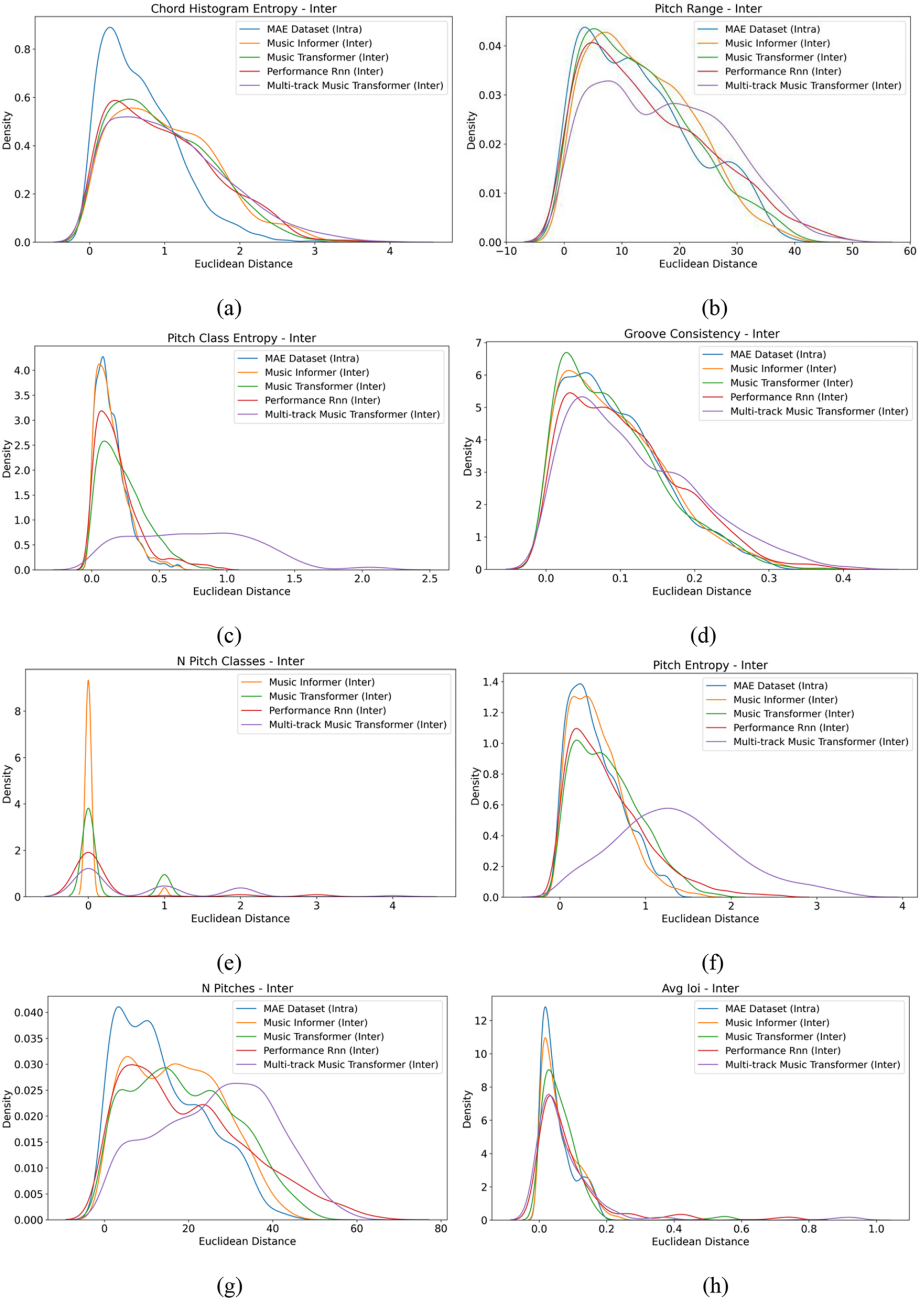
An example of inter-set distance for MAESTRO Dataset, Music Informer, Music Transformer, Performance RNN, and Multi-track Music Transformer. The metrics displayed are: (**a**) CHE, (**b**) PR, (**c**) PCE, (**d**) GC, (**e**) NPC, (**f**) PE, (**g**) NP and (**h**) AIOI.

The histograms of Euclidean inter-set distances for these features from both the dataset and model-generated samples are displayed in Fig. 9. It can be concluded that the generated sample distribution of Music Informer aligns closely with MAESTRO dataset across multiple features, demonstrating exceptional performance particularly in PCE, GC, PE, and AIOI. This fully demonstrates the effectiveness of our model.

Table 3 presents the results for the KLD and OA metrics, clearly indicating that Music Informer surpasses the other three baseline models in several metrics. Specifically, Music Informer exhibits the lowest KLD values in AIOI and GC, reflecting a closer alignment of its generated samples with the distribution of the real dataset in these two features. Additionally, higher OA values in features NP, PCE, AIOI, GC, and PE demonstrate a greater overlap in feature distribution between the generated samples of Music Informer and the real dataset. These findings suggest that Music Informer excels in capturing and replicating the features of the real dataset, resulting in compositions that more closely resemble the real dataset in terms of both musicality and feature distribution. Please note that the NPC feature in the MAESTRO dataset has a fixed value of 12, resulting in no meaningful distribution to compare. As such, KLD and OA values are not applicable for this feature and are represented as—in this table.

**Table 3:** KLD and OA results comparing samples generated by music informer, music transformer, performance RNN, and multi-track music transformer to the MAESTRO dataset.

	Music transformer	Performance RNN	Multitrack music transformer	Music informer	
PR	KLD	**0.0174**	0.0350	0.0399	0.0354
	OA	**0.9056**	0.8802	0.8102	0.8838
NP	KLD	0.0400	**0.0127**	0.1639	0.0456
	OA	0.7854	0.7922	0.5952	**0.8189**
PCE	KLD	0.1734	0.1536	**0.0398**	0.0813
	OA	0.8713	0.9372	0.4143	**0.9661**
AIOI	KLD	2.2400	0.5632	2.3059	**0.0646**
	OA	0.7803	0.7326	0.7480	**0.8688**
CHE	KLD	0.0283	0.0475	**0.0256**	0.0304
	OA	**0.7725**	0.7579	0.7283	0.7472
GC	KLD	0.0698	0.0256	0.0132	**0.0081**
	OA	0.9253	0.8924	0.8340	**0.9380**
PE	KLD	**0.0271**	0.1469	0.1182	0.0774
	OA	0.8136	0.8330	0.3552	**0.9075**
NPC	KLD	–	–	–	–
	OA	–	–	–	–

The bold indicates the optimal results of KLD and OA value of different evaluation metrics with different models.

### B. Subjective results

Since the results of objective metrics can only serve as a reference and cannot reflect the overall quality of music, we conducted subjective evaluation, which is the indispensable and most convincing evaluation method. Ten samples were randomly generated for each of the four models: Music Informer, Music Transformer, Performance RNN, and Multi-track Music Transformer. In the initial phase of the experiment, five participants participated to test the evaluation process. To ensure the statistical significance and reliability of the results, we expanded the sample size to 30 participants (15 male and 15 female), aged 18–30 years, recruited through university advertisements and online platforms. Among the participants, 20% had prior experience in learning musical instruments or vocals, and all participants provided informed consent before the experiment.

Each participant listened to all 40 samples in a randomized order using high-quality headphones in a quiet environment and rated them on a 5-point Likert scale (1 = low, 5 = high) based on three subjective metrics:

1. Coherence (C), which refers to the structural consistency and logical flow of the sample;

2. Naturalness of pitch(N), which measures how human-like and smooth the sample sounds;

3. Overall Quality (Q), which reflects the general impression of the sample, including its appeal and enjoyment.

**Fig. 10:**
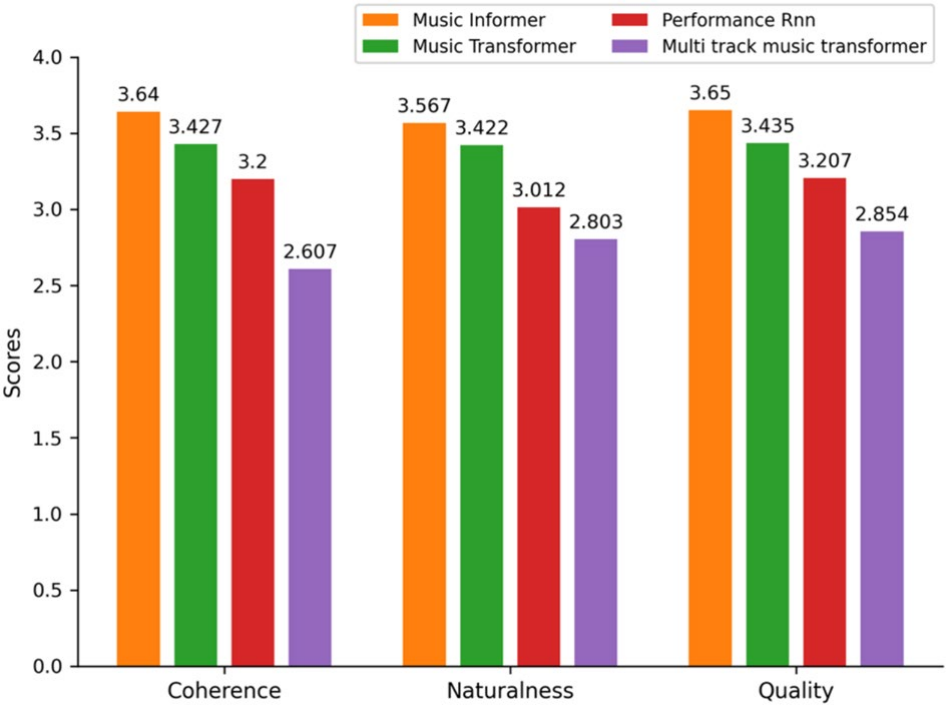
Subjective evaluation results of the samples generated by Music Informer, Music Transformer, Performance RNN, and Multi-track Music Transformer.

The evaluation results, presented in Fig. 10, show that Music Informer surpasses the three baseline models in the C, N, and Q, demonstrating the effectiveness of the model and aligning with the objective results. By the Mann–Whitney U test, we further verify that the samples generated by Music Informer and Music Transformer exhibit significant performance differences in terms of C and Q. Specifically: For C, the test yielded a U statistic of 33.00 with a* p*-value of 0.0152, indicating a significant difference (*p *< 0.05).For Q, the U statistic was 31.00 with a* p*-value of 0.0435, also indicating a significant difference (*p *< 0.05).These results demonstrate that Music Informer significantly outperforms Music Transformer in generating music with higher coherence and overall quality.

Both subjective and objective evaluations reveal that the Music Informer significantly outperforms the Music Transformer, Performance RNN, and Multi-track Music Transformer. The samples generated by Music Informer exhibit higher coherence, naturalness and overall quality.

## Conclusions and future work

Music Informer, a music generation model, features a decoder with three layers of probabilistic sparse self-attention and three layers of relative local attention, totaling six layers. An LSTM structure with two LSTM layers follows the decoder, culminating in the final output. Experimental results demonstrate the Music Informer’s superior training performance, evidenced by higher accuracy and lower loss, reflecting its effectiveness in learning and predicting music data.

Music samples generated by the Music Informer outperform those from the Music Transformer, Performance RNN, and Multi-Track Music Transformer across metrics such as PCE, NPC, PE, NP, and AIOI. These samples closely resemble the characteristics of the MAESTRO dataset, showcasing the model’s ability to better capture musical features and generate more melodic compositions. Furthermore, the Music Informer conserves 21.73%, 31.87%, and 41.33% of computational resources compared to the Music Transformer, Performance RNN, and Multi-Track Music Transformer. This efficiency is attributed to the ProbSparse self-attention and the LSTM structure, which capture long-term dependencies to maintain musical continuity and consistency. Relative local attention enhances the understanding and of local music patterns and structures, improving accuracy and coherence in music generation. The integration of ProbSparse self-attention, relative local attention, and the LSTM structure enables the Music Informer to comprehensive capture music features and patterns, enhancing the coherence and overall quality of generated music.

Despite these advancements, several limitations persist. A significant challenge lies in the evaluation of the “musicality” of the generated compositions. Musicality is a subjective concept that is difficult to quantify, as it involves complex factors such as emotional expression, thematic development, and structural coherence. While subjective listener studies provide valuable insights into the perceived quality of the generated music, their outcomes are often influenced by individual preferences and cultural contexts, limiting their generalizability. On the other hand, objective metrics, such as CHE, though useful for quantitative analysis, may not fully capture the subtle and nuanced characteristics that define musicality. To address these challenges, future research should focus on developing more comprehensive evaluation frameworks that integrate subjective and objective approaches, enabling a more holistic and reliable assessment of the musical quality of generated compositions.

Beyond evaluation challenges, future research should also aim to enhance the diversity of training samples to better understand different music styles and instruments, improve feature extraction techniques for complex attributes like dynamic changes and expressiveness, and expand research to encompass a wider variety of music types and applications, thereby boosting the model’s generalization capabilities.

## Data Availability

The MAESTRO dataset used in this paper can be downloaded from the following website: https://magenta.tensorflow.org/datasets/maestro. The source code for our Music Informer is available at the following website: https://github.com/1Garlicboy/MusicInformer. Recordings generated by Music Informer can be accessed at https://github.com/1Garlicboy/MusicInformer/tree/main/samples.
